# Detection of Particulate Matters with a Field-Portable Microscope Using Side-Illuminated Total Internal Reflection

**DOI:** 10.3390/s21082745

**Published:** 2021-04-13

**Authors:** Haechang Yang, Sanghoon Shin, Dongmin Seo, Jaewon Park, Sungkyu Seo

**Affiliations:** 1 Department of Electronics and Information Engineering, Korea University, Sejong 30019, Korea; didgockd123@korea.ac.kr (H.Y.); ghost10s@korea.ac.kr (S.S.); 2 Maritime Safety Research Division, Korea Research Institute of Ships & Ocean Engineering, Daejeon 34103, Korea; dseo@kriso.re.kr; 3 School of Microelectronics, Southern University of Science and Technology, Shenzhen 518055, China

**Keywords:** particulate matter (PM), optical detection, side illumination, total internal reflection (TIR), field-portable microscope

## Abstract

Field-portable observation and analysis of particulate matter (PM) is required to enhance healthy lives. Various types of the PM measurement methods are in use; however, each of these methods has significant limitations in that real time measurement is impossible, the detection system is bulky, or the measurement accuracy is insufficient. In this work, we introduce an optical method to perform a fast and accurate PM analysis with a higher-contrast microscopic image enabled by a side-illuminated total internal reflection (TIR) technique to be implemented in a compact device. The superiority of the proposed method was quantitatively demonstrated by comparing the signal-to-noise ratio of the proposed side-illuminated TIR method with a traditional halogen lamp-based transmission microscope. With the proposed device, signal-to-noise ratios (SNRs) for microbeads (5~20 µm) and ultrafine dust particles (>5 µm) increased 4.5~17 and 4~10 dB, respectively, compared to the conventional transmission microscope. As a proof of concept, the proposed method was also applied to a low-cost commercial smartphone toy microscope enabling field-portable detection of PMs. We believe that the proposed side-illuminated TIR PM detection device holds significant advantages over other commonly used systems due to its sufficient detection capability along with simple and compact configuration as well as low cost.

## 1. Introduction

Ever increasing particulate matter (PM), released from factories, automobiles, and thermal power plants, affects human health in various ways when absorbed into the human body through the respiratory tract and skin [1]. Short-term exposure to air pollutants is associated with bronchial diseases such as chronic obstructive pulmonary disease, cough, and asthma. Long-term exposure to air pollution can cause diabetes or even cause heavy metals to be accumulated in the bones of the human body resulting in neurological or cardiovascular related diseases [2,3,4]. To scope with those exposures by informing PM level, several technologies have been developed and utilized. Mass concentration, β-ray absorption, light scattering, and tapered element oscillating microbalances (TEOM) are commonly used methods for measuring PM; however, these methods have certain limitations. While the mass concentration method, β-ray absorption method, and TEOM method can provide accurate results, they require bulky systems and, thus, are not easy to make them portable. Furthermore, the mass concentration method requires 24 h of sampling time that needs to be done manually and β-ray absorption lacks stability in separation of the PM samples depending on the sample viscosity. The light scattering method allows a portable system to be built, but it suffers from significant errors in the process of converting the particle counts to mass concentration, which is indirect estimation of PM, after detecting fine dust [5,6,7]. While low-cost commercial off-the-shelf (COTS) PM sensors also have been widely developed [8,9,10,11], they still employed the indirect light scattering approach.

In this work, we present a field-portable direct PM measurement system using a side-illuminated total internal reflection (SI-TIR) microscopy technique. The system consists of light emitting diodes (LEDs) and a PMMA (Poly methyl methacrylate) plate, having LED light sources attached to four sides. The PMMA plate functions as an optical waveguide and the size of PM on the PMMA plate can be directly measured from the high-contrast microscopy images by calculating signal-to-noise ratios (SNRs) of each particle. When combined with a commercially available smartphone camera microscope, the developed system can be used as a portable PM detector to be used on sites.

## 2. Materials and Methods

### 2.1. PM Detection System with Side-Illuminated Total Internal Reflection

**Figure 1:**
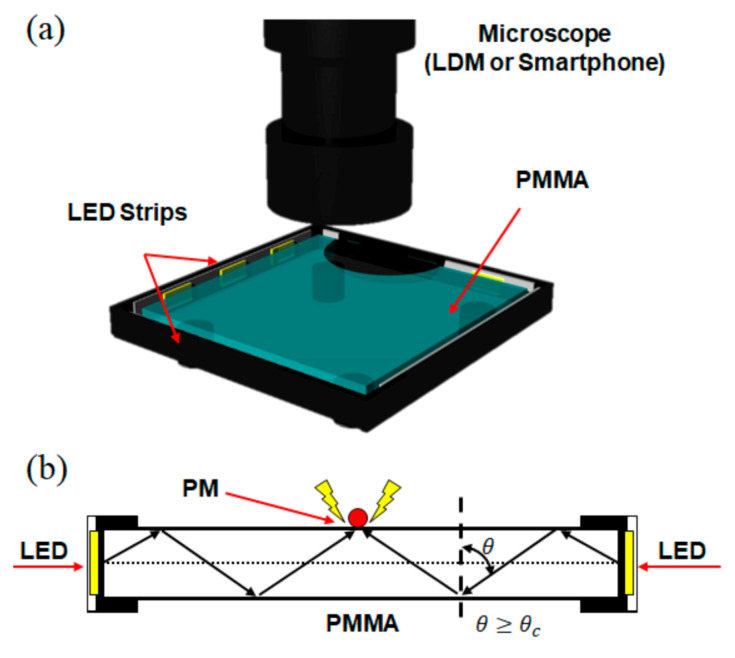
Schematics of the developed side illumination total internal reflection (TIR)-based particulate matter (PM) detection system. (**a**) Microscope (long-distance microscope (LDM) or smartphone-compatible toy microscope) can directly image the PM on the PMMA (Poly methyl methacrylate) plate with high contrast by side-illuminated TIR. (**b**) Incident LED light (θ) over the critical angle ($\theta_{c})$ is confined inside the PMMA plate by TIR effect, enabling high-contrast microscopic imaging.

The SI-TIR-based PM detection system is composed of a PMMA plate (50 × 50 × 5 mm) attached with four white 2835 LED strips (12 V, 20 μW) on each side. Side illumination generates TIR within the PMMA plate. When PMs settle on the PMMA surface, the critical angle of the light traveling inside the PMMA plate changes due to the refractive index difference between the PM on the PMMA surface and the air, resulting in light scattering. The light scattering allows us to acquire high-contrast microscopy images for measuring the size and the quantity of the PM. The schematic of the developed side illumination TIR-based PM detection system (Figure 1a) and the working principle (Figure 1b) are shown in Figure 1.

### 2.2. Finite-Differential Time Domain Simulation

The finite-difference time domain (FDTD) is a numerical analysis technique employed to solve the Maxwell equations enabling visualization of the electromagnetic fields of the proposed optical design. FDTD simulation for side-illuminated TIR-based PM detection system was performed using an RSoft FullWAVE (Synopsys, Mountain View, CA, USA). The simulation was designed with spherical polystyrene beads (refractive index: 1.606) of three different sizes (5, 10, and 20 μm) on a PMMA plate (refractive index: 1.49). The illumination was set at the four sides of the PMMA plate [12] to closely mimic the developed system.

### 2.3. Image Acquisition

Three different image acquisition configurations were tested for comparison; the traditional illumination, i.e., transmission, with a long-distance microscope (LDM), the side-illuminated TIR with an LDM, and the side-illuminated TIR with a smartphone microscope. For the LDM image acquisition, an LDM (K2/SC CF-3, Infinity, Centennial, CO, USA) equipped with a CMOS digital camera (EYECam, Brunel Microscope, Wiltshire, UK) was used, and light was illuminated either using a halogen lamp (20 μW), placed below the PMMA plate (transmission mode) or LED strips attached to the PMMA plate (side-illuminated TIR mode). The power of the LED for side illumination was set to match with the halogen lamp (20 μW) used for the transmission illumination.

For the portable configuration, same-side illumination condition as LDM was used, but images were acquired using a smartphone (iPhone 6S, Apple, Cupertino, CA, USA) assembled with a smartphone toy microscope, which costs less than USD 5 in a retail store (Smartphone Microscope, DAISO, Sejong, Korea).

### 2.4. Particle Preparation

Detection and quantification of PM using the developed system was demonstrated using polystyrene beads (Duke Standards 2000 series, Thermo Fisher Scientific, Waltham, MA, USA) of three different sizes (5, 10, and 20 μm) suspended in an aqueous solution and ISO 12103-1 A1 ultrafine test dusts (Powder Technology, Arden Hills, MN, USA). The polystyrene beads were diluted in de-ionized water, and the bead solution was pipetted on a microscope cover slip. Then, it was dehydrated using a hot plate at the temperature of 50 °C before the dried beads were randomly dispensed on top of the PMMA plate. The solid ultrafine dusts were directly dispensed on top of the PMMA plate in a closed dust box. 

### 2.5. Signal-to-Noise Ratio (SNR) Analysis

To quantify the contrast enhancement of the proposed platform, SNR of the detected signals was calculated using the below Equation (1) [13].

(1) $$\mathrm{SNR}\;\mathrm{dB}\;=\;20\log\left|\frac{\mathrm{MAX}\left(I\right){\;-\;\mu }_{b}}{\sigma_{b}}\right|$$

where $\mathrm{MAX}\left(I\right)$ is the light intensity of particle, $\mu_{b}$ is the mean value of the background noise region, and $\sigma_{b}$ is the variance of the background noise region.

## 3. Results and Discussions

### 3.1. Simulation of the Side-Illuminated PM Detection

**Figure 2:**
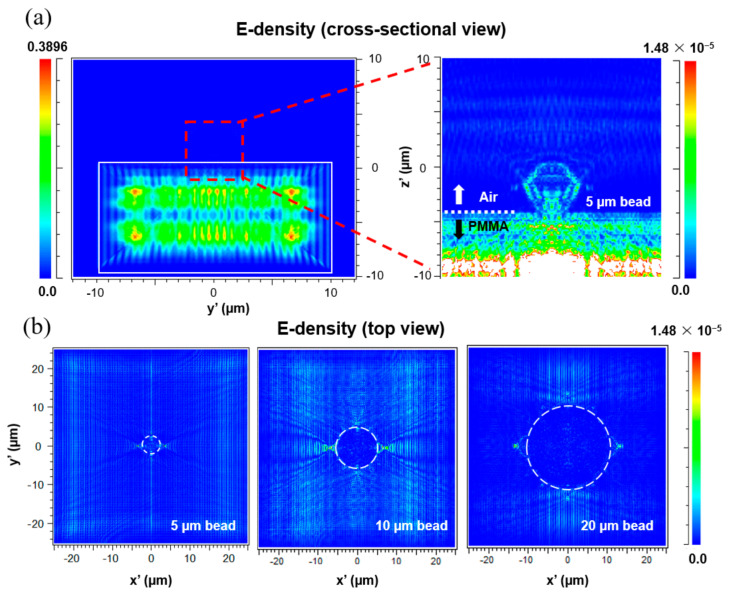
Electromagnetic field distribution of the side−illuminated TIR platform. Energy density plots from cross−sectional view (**a**) and top view (**b**). The TIR effect could be well observed inside of the PMMA plate, and four boundaries of microbeads showed concentrated energy compared to the other regions.

The TIR phenomenon occurs when the angle of incidence is larger than the critical angle. For the simulation, the refraction angle was calculated according to the incident angle of the light on the PMMA substrate from the four external LED light sources by Snell’s law. First, the transmission angle of the light source entering the PMMA substrate from the LEDs was obtained using the equation $n_{i}{\sin\theta }_{i}{\;=\;n}_{t}{\sin\theta }_{t}$ [14], where n_i_ is the refractive index of incident medium (PMMA), and n_t_ is the refractive index of transmitting medium (air). Refractive index of air and the PMMA was set to 1 and 1.49, respectively. The critical angle of the system for TIR was then found from $\theta_{c}=\arcsin{(n}_{t}{/n}_{i}$). By using this simple procedure, transmission and critical angles were set to find the launching angle of the illumination in the simulation (λ = 550 nm). Electromagnetic field distribution of the side-illuminated TIR platform is described in Figure 2. Given in energy density plots from cross-sectional view (Figure 2a) and top view (Figure 2b), the TIR effect could be well observed inside of the PMMA plate, and four boundaries of microbeads showed concentrated energy compared to the other regions.

### 3.2. Experiment of the Side-Illuminated TIR PM Detection

**Figure 3:**
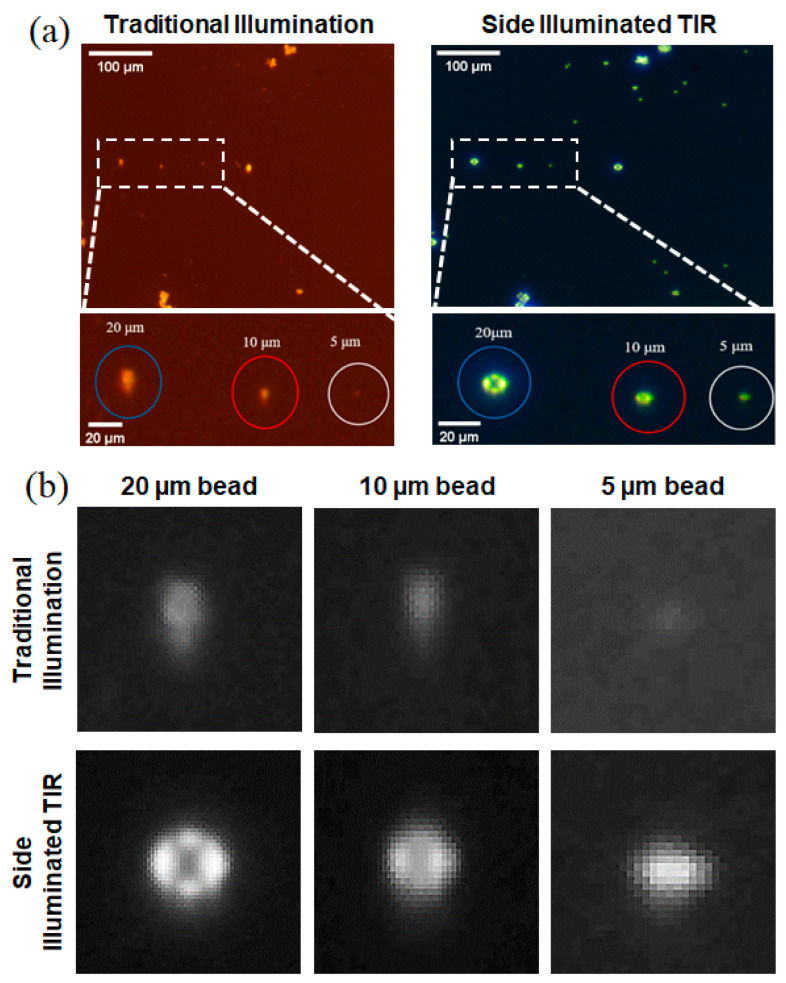
Images of polystyrene beads acquired using an LDM with two different illumination methods. Transmission illumination is blurrier and shows a smaller number of polystyrene beads when compared to side-illuminated TIR. (**a**) Raw images and (**b**) digitally zoomed images from each method.

Figure 3 shows the images of polystyrene beads acquired using an LDM with two different illumination methods. It can be seen that the transmission illumination is blurrier and shows a smaller number of polystyrene beads when compared to the side-illuminated TIR. In particular, 5 μm beads could be hardly identified for the transmission illumination image, while it could be clearly detected when using the side-illuminated TIR. One interesting thing to note is that the simulation results in Figure 2b show four peak signal points on the bead surface at the direction where the LEDs are attached, i.e., four sides of the PMMA plate, and the same peaks could be seen in actual images taken with the side-illuminated TIR system (Figure 3). Four peak points were significantly noticeable for 20 and 10 µm beads, while individual peaks are not distinguishable for a 5 µm bead, although the bead is clearly detectable. We believe this allowed us to take high-contrast images and have enhanced the detection of smaller particles for our side-illuminated TIR PM detection system in comparison to the conventional transmission illumination method. Likewise, the side-illuminated TIR system yielded more clear and identifiable images when the same experiment was done using ISO 12103-1 A1 ultrafine test dusts (Supplementary Figure S1).

**Figure 4:**
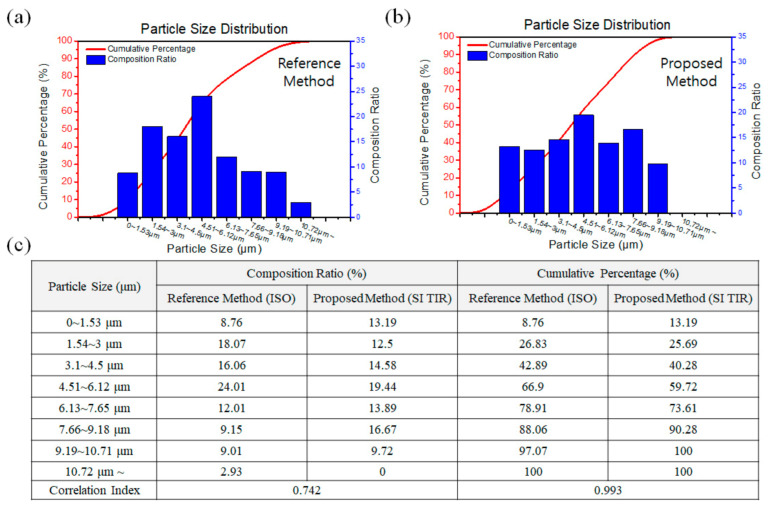
Analysis of particle size distribution using the ISO 12103-1 A1 ultrafine test dust. Actual particle size distribution of the tested sample from the datasheet (**a**) and data analyzed using the developed system (**b**). Correlation index between the reference and proposed method was calculated as 0.742 for the composition ratio and 0.993 for the cumulative percentage, respectively (**c**).

Next, analysis of particle size distribution was carried out using the ISO 12103-1 A1 ultrafine test dust. Figure 4a shows the actual particle size distribution of the tested sample from the datasheet, and Figure 4b shows the data analyzed using the developed system. Due to the resolution limit of the LDM microscope (1.6 µm), particles smaller than 1.6 µm could not be differentiated, but the system successfully analyzed the size distribution up to 10.71 µm. A total of 144 particles were analyzed, and the cumulative percentage curve of the analyzed data showed a similar trend with the reference datasheet. It should be noted that the experiment was done using a low-resolution LDM microscope (×3.05 objective and ×10 eyepiece), and the accuracy as well as the detection limit can be further enhanced if a higher-resolution microscope is employed. Using this low-resolution microscope, e.g., ×30.5 magnification in total, the correlation index between the reference and proposed method was calculated as 0.742 for the composition ratio and 0.993 for the cumulative percentage, respectively (Figure 4c). In addition, when the developed system is used as a portable system, rough estimations that the system can provide are often sufficient for on-site detection.

### 3.3. Quantitative Image Comparison

**Figure 5:**
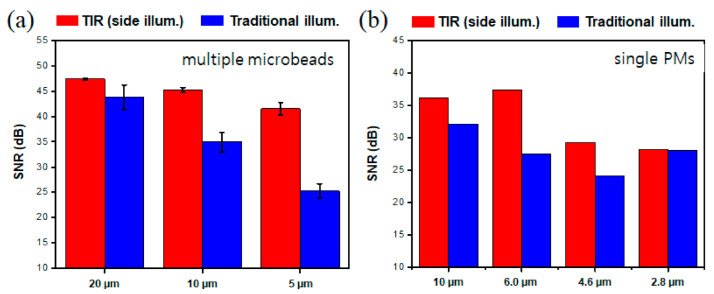
Signal-to-noise ratio (SNR) comparison for each method. SNR analysis of polystyrene bead of three different sizes (5, 10, and 20 μm) between two different illumination methods (**a**). SNR analysis of ISO 12103-1 A1 ultrafine test dusts of four different sizes (10, 6.0, 4.6, and 2.8 μm) between two different illumination methods (**b**).

Acquired images were further investigated by analyzing SNR of images for quantitative comparison. Figure 5a shows the SNR analysis of polystyrene beads of three different sizes between two different illumination methods. In all cases, the side-illuminated TIR resulted in higher SNR compared to the transmission illumination using a halogen lamp. In particular, the side-illuminated TIR was more effective in detecting smaller particles. SNR for the 20 µm beads was only 4.5 dB higher, while it was 17 dB higher for 5 µm beads when compared to the transmission illumination, respectively. The side-illuminated TIR showed higher SNR for the ISO 12103-1 A1 ultrafine test dust as well. As the test dust contains particles of various sizes, we have chosen four different size particles (10, 6.0, 4.6, and 2.8 μm) for comparison (Figure 5b). For the images of 10, 6.0, and 4.6 μm particles, the side illumination TIR system showed from 4 to 10 dB higher SNR compared to the transmission illumination, yet no difference could be seen for 2.8 μm. These results demonstrate well the capability of the developed system for detecting smaller particles over conventionally used transmission-based microscopy techniques.

### 3.4. Field-Portable Microscope for PM Detection Using Smartphone

**Figure 6:**
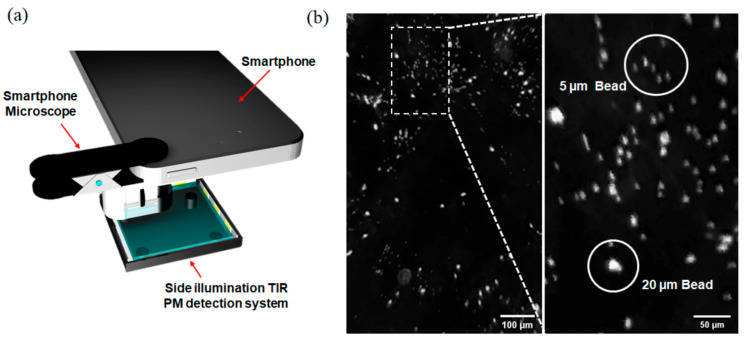
Side-illuminated TIR PM detection system paired with smartphone. Configuration of the smartphone camera attached with a smartphone toy microscope (**a**) and captured images from it (**b**).

Simple and small configuration of the side-illuminated TIR PM detection system allowed it to be used as a portable system when paired with a smartphone. As shown in Figure 6a, images were acquired with the smartphone camera attached with a smartphone toy microscope, which costs less than USD 5 in a retail store (Smartphone Microscope, DAISO, Korea). The side-illuminated TIR system successfully generated strong scattering of microbeads, and high-contrast images could be acquired without any problem using a smartphone (Figure 6b). To our surprise, 5 µm polystyrene beads, which were hard to detect even with LDM in the transmission illumination methods, could also be detected with our portable setup (Figure 6b—inset). This result clearly demonstrates that the developed system can be used as a portable setup for on-site detection of PM at extremely low cost compared to conventional systems. It also shows the potential of our system to be used in various other applications that require the detection of small analytes on site.

## 4. Conclusions

We have developed a simple, compact, and low-cost system for detecting micro particles and PM. The system utilizes a side-illuminated total internal reflection technique that uses a PMMA plate as a wave guide. The refraction index difference between the analyte, e.g., micro particles or PM, and air enables it to acquire high-contrast microscopy images for particle detection. Enhanced detection capability of the developed system was validated by the electromagnetic field simulation as well as the experimental results using polystyrene microbeads and ultrafine test dust kit. When compared to the conventional transmission illumination, advantages of the proposed method were that higher-contrast images could be acquired with the developed system, and micro particles could be detected better with higher SNRs, i.e., 4.5~17 dB for microbeads (5~20 µm) and 4~10 dB for ultrafine dust particles (>5 µm). In addition, the system could be also utilized as a portable system by employing a smartphone-compatible toy microscope (<5 USD). Even with this low-cost portable setup, particles as small as 5 µm could be clearly imaged and detected. The developed portable system showed significant limitation in detecting particles smaller than 5 µm due to the resolution limit of the toy microscope used in this study; however, this can be further enhanced with a higher-resolution imaging system. We believe that our side-illuminated TIR PM detection system holds great promise over other commonly used PM detection systems due to its sufficient detection capability along with simple and compact configuration as well as low cost, especially for the resource-limited settings.

## Data Availability

Not applicable.

## Supplementary Materials

The following are available online at https://www.mdpi.com/article/10.3390/s21082745/s1.
