# Supplementary material for: Detection of Particulate Matters with a Field-Portable Microscope Using Side-Illuminated Total Internal Reflection
Source: Sensors (Basel). 2021 Apr 13;21(8):2745. doi: 10.3390/s21082745 (PMC8070112; doi:10.3390/s21082745)

Supplementary Material

Detection of particulate matters with a field-portable microscope using side illuminated total internal reflection

Haechang Yang[1,], Sanghoon Shin[1,*], Dongmin Seo[2,*], Jaewon Park[3,†], and Sungkyu Seo[1,†]*

[1] Department of Electronics and Information Engineering, Korea University, Sejong, Republic of Korea

[2] Maritime Safety Research Division, Korea Research Institute of Ships & Ocean Engineering, Daejeon, Republic of Korea

[3] School of Microelectronics, Southern University of Science and Technology, Shenzhen, China

* These authors equally contributed to this study.

† Correspondence to:

(S. Seo) E-mail; sseo@korea.ac.kr, Tel; +82-44-860-1427, Fax; +82-44-860-1585, Address; #415, Accelerator and ICT Bldg., Korea University, Sejong 30019, Republic of Korea

(J. Park) Email; jwpark@sustech.edu, Tel; +86-755-8801-8574, Address; Southern University of Science and Technology, 1088 Xueyuan Avenue, Shenzhen, Guangdong, 518055, China

Figure S1. Side illuminated TIR system yielded more clear and identifiable images than traditional illumination when same experiment was done using ISO 12103-1 A1 ultrafine test dusts.

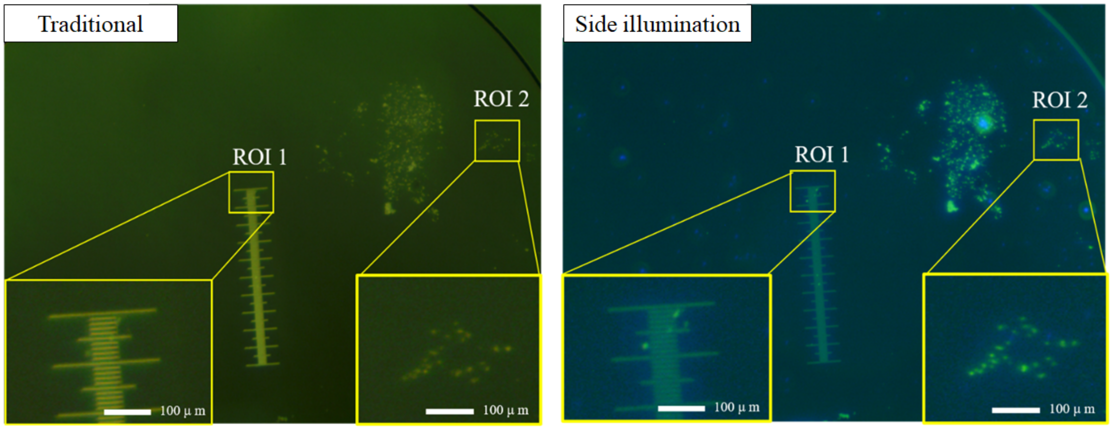

Supplement: Supplementary file 1 [file sensors-21-02745-s001.pdf]
